# Four new species of Cerambycidae (Coleoptera) from Paraguay

**DOI:** 10.3897/zookeys.507.9277

**Published:** 2015-06-08

**Authors:** Ole Mehl, Maria Helena M. Galileo, Ubirajara R. Martins, Antonio Santos-Silva

**Affiliations:** 1 PPG Biologia Animal, Departamento de Zoologia, Universidade Federal do Rio Grande do Sul, Porto Alegre, RS, Brazil; 2 Museu de Zoologia, Universidade de São Paulo, São Paulo, São Paulo, Brazil; 3 Fellow of the Conselho Nacional de Desenvolvimento Científico e Tecnológico

**Keywords:** Cerambycinae, key, Lamiinae, South America, taxonomy

## Abstract

Four new species of Cerambycidae are described from Paraguay: *Eranina tomentilla* (Hemilophini); *Mimasyngenes concolor* (Desmiphorini); *Recchia drechseli* (Aerenicini); and *Microibidion bimaculatum* (Neoibidionini). The new species are included in known keys.

## Introduction

Bates (1866) described *Erana* to include a single species: *Erana cincticornis* Bates, 1866. Still in the 19th Century, Bates (1874, 1881, 1885) described nine other species in this genus. In the 20th Century, 14 species were described, and one was transferred to *Erana*. More recently, in this century, 12 new species were described. Monné (2005) proposed *Eranina* to replace *Erana* Bates, 1866, preoccupied by *Erana* Gray, 1840 (Aves). Currently *Eranina* includes 36 species, distributed in North (Mexico), Central and South America. Martins and Galileo (2014) reviewed the 18 South American species and provided a key to them.

*Mimasyngenes* Breuning, 1950 includes 14 species, apparently occurring only in South America. A single species was recorded for Costa Rica (*Mimasyngenes icuapara* Galileo & Martins, 1996) by Swift et al. (2010), but Monné (2014) considered the record as doubtful: “Costa Rica ?, Brazil (São Paulo), Argentina (Misiones).” Clarke (2007) revised the species of *Mimasyngenes* occurring in Bolivia, and provided a key to the ten species known at that time. After the publication of that key, four new species were described in *Mimasyngenes*.

*Recchia* Lane, 1966 is a predominantly South American genus, with a single species also occurring in Central America: *Recchia hirsuta* (Bates, 1881). Currently *Recchia* includes 22 species. From those species, seven were transferred from *Aerenica* Dejean, 1835, of which two were originally described in *Saperda* Fabricius, 1775. Galileo and Martins (1992) synonymized *Trichohippopsides* Breuning, 1980 with *Recchia*, and the type species of the former, *Trichohippopsides albicans* Breuning, 1980, with *Recchia albicans* (Guérin-Méneville, 1844). Martins and Galileo (1998) considered *Coruparana* Lane, 1966 as a new synonym of *Recchia*. Thus, two more species were added to *Recchia*.

*Microibidion* Martins, 1962 is exclusively South American, and includes six species. The latest species was described from Bolivia 43 years ago. Martins (2009) revised the species of this genus and provided a key to them.

## Material and methods

Photographs were taken with Canon EOS Rebel T3i DSLR camera, Canon MP-E 65mm f/2.8 1–5× macro lens, controlled by Zerene Stacker AutoMontage software.

The collection acronyms used in this study are as follows:

MZSP Museu de Zoologia, Universidade de São Paulo, São Paulo, Brazil.

## Systematics

### Lamiinae Latreille, 1825

#### Hemilophini Thomson, 1868

##### Eranina tomentilla sp. n.

Taxon classificationAnimaliaColeopteraCerambycidae

http://zoobank.org/ACFC97FC-8369-4A8F-AC34-02AA1591385C

Figs 1 , 2 , 3

###### Description.

Integument black; the following parts orange: frons, most of clypeus, most of lateral side of mandibles, maxillary palpomeres I–III, base of maxillary palpomere IV, ventral side of head, area under lower eye lobes, base of antennomere III, base and nearly all ventral side of antennomere IV, base of antennomeres V–VII, large central “V-like” area on pronotum, parts of lateral side of prothorax, lateral longitudinal band on basal half of elytra (reaching lateral margin on basal fourth), basal two-thirds of profemora, basal half of mesofemora, basal third of metafemora; the following brown-yellowish: vertex, about central two-fourths of dorsal side of antennomere III; parts of lateral side of prothorax; tarsal claws; brown longitudinal band on basal half of elytra, between orange band and black area; ventral side of scape dark-brown.

Head. Frons transverse, coarse, abundantly punctate (most punctures obliterated by pubescence); pubescence yellow, dense, mixed with long, sparse setae. Coronal suture well-marked from clypeus to anterior edge of prothorax. Area between antennal tubercles depressed, coarse punctate; pubescence sparser than on anterior region of frons; laterally with long setae. Antennal tubercles with yellow pubescence on base, dark-brown on remaining surface; with long, abundant setae. Vertex with yellow, dense pubescence. Area behind lower eye lobes coarse, abundantly punctate; pubescence, moderately dense on narrow band close to eyes, very sparse towards anterior edge of prothorax. Genae with yellow pubescence, mixed by long, sparse setae. Distance between upper eye lobes equal to 0.2 times the length of scape; distance between lower eye lobes, in frontal view, equal to 0.6 times the length of scape. Antennae as long as 1.2 times the elytral length; almost reaching elytral apex; scape, pedicel and antennomere III with abundant, moderately long erect setae, mixed with also abundant very long setae throughout; antennomere IV with setae as on III, but sparser; antennomeres V–XI dorsally with sparse, moderately long setae, ventrally with very long, sparse setae; antennal formula based on antennomere III: scape = 0.77; pedicel = 0.14; IV = 0.44; V = 0.25; VI = 0.23; VII = 0.21; VIII = 0.21; IX = 0.19; X = 0.19; XI = 0.21.

Thorax. Prothorax cylindrical, transverse. Pronotum moderately coarse, abundantly punctate; pubescence yellow on area with orange integument, directed towards center, forming elongated tuft; on each side of anterior half, spot of yellowish-white pubescence; remaining surface with very short, slightly conspicuous brownish-yellow pubescence; with long, sparse setae. Lateral sides of prothorax moderately coarse, abundant punctate; pubescence brownish-yellow, dense on areas with light integument, brown on areas with dark integument. Metasternum laterally pubescent, very sparsely pubescent towards middle. Elytra: coarse, abundantly punctate on basal half, gradually finer, sparser punctate towards apex; pubescence dense, very conspicuous on area with orange integument, dark-brown, very short on remaining surface; with moderately long, abundant erect setae throughout; apex individually rounded. Legs: femora pubescent, with long, moderately abundant setae (mainly ventrally).

Abdomen. Ventrites I–IV laterally pubescent, distinctly sparser towards center, interspersed with long, sparse setae. Ventrite V trapezoidal, 1.5 times as long as IV, with long sparse setae, distinctly denser near apex, laterally pubescent; apex rounded.

###### Type material.

Holotype female: PARAGUAY, San Pedro: La Florida (56°59'W, 24°29'S), 27.IV.2012, U. Drechsel col. (MZSP).

###### Dimensions in mm (female).

Total length, 8.0; length of prothorax at center, 1.2; anterior width of prothorax, 1.3; posterior width of prothorax, 1.4; humeral width, 1.9; elytral length, 5.8.

###### Etymology.

Latin, *tomentum* = pubescence consisting of moderately long, soft, entangled hairs; *illa* = suffix, added to feminine nouns to denote a diminutive form. Relating to the hairy look of the species.

###### Remarks.

*Eranina tomentilla* sp. n. differs from *Eranina argentina* (Bruch, 1911) as follows (comparison with syntype female deposited at MZSP): distance between upper eye lobes equal to 0.2 times the length of scape; setae on antennomere III distinctly more abundant; antennomere III 2.2 times longer than IV; basal integument of antennomeres V–VII orange. In *Eranina argentina* the distance between upper eye lobes is equal to 0.35 times length of scape, the setae on antennomere III are sparser (mainly the shorter ones), antennomere III is 1.7 times as long as IV, and antennomeres V–VII are entirely dark. It can be separated from females of *Eranina porangaba* (Galileo & Martins, 1998) by antennae not reaching elytral apex (surpassing in *Eranina porangaba*), and by antennomere III longer than twice length of IV (distinctly shorter than twice the length of IV in *Eranina porangaba*).

*Eranina tomentilla* can be included in the alternative of couplet “16”, from Martins and Galileo (2014) (translated; couplet 15 modified):

**Table:** 

15(13)	Elytra without yellow macula on humeri; antennomere IV white with basal ring black. French Guiana, Brazil (Amazonas)	***Eranina cincticornis* (Bates, 1866)**
–	Elytra with yellow macula on humeri; antennomere IV basally yellowish	**16**
16(15)	Elytra mostly yellowish with circum-scutellar region and distal third reddish-brown; antennomere III shorter than twice the length of IV; antennomeres V–VII entirely dark. Paraguay, Argentina	***Eranina argentina* (Bruch, 1911)**
–	Elytra mostly dark; antennomere III longer than twice the length of IV; antennomeres V–VII orange on base. Paraguay	***Eranina tomentilla* sp. n.**

#### Desmiphorini Thomson, 1860

##### Mimasyngenes concolor sp. n.

Taxon classificationAnimaliaColeopteraCerambycidae

http://zoobank.org/33412AE4-55CE-4D84-B1C5-D8289CD33A88

Figs 4 , 5 , 6

###### Description.

Integument black, with labrum and palpi reddish-brown; pubescence white; setae dark-brown.

Head. Frons transverse, coarse, moderately abundantly punctate; pubescence sparse, distinctly not obliterating integument, slightly more concentrated along coronal suture and margin of eyes; setae long, sparse. Sculpture and pubescence of vertex as on frons. Coronal suture marked from clypeus to anterior edge of prothorax. Area behind eyes with sparse pubescence. Genae with sparse pubescence towards clypeus, glabrous towards apex. Distance between upper eye lobes equal to 0.6 times the length of scape; distance between lower eye lobes, in frontal view, equal to 0.8 times the length of scape. Antennae as long as 1.3 times the elytral length; reaching elytral apex; antennal segments sparsely pubescent; scape, pedicel and antennomeres III–VII with sparse, very long setae throughout (ventrally longer and more abundant on antennomeres); antennomeres VIII–X with moderately long setae near apex; antennal formula based on antennomere III: scape = 0.96; pedicel = 0.33; IV = 1.08; V = 0.75; VI = 0.75; VII = 0.67; VIII = 0.58; IX = 0.46; X = 0.42; XI = 0.42.

Thorax. Prothorax transverse, distinctly wider between lateral tubercles and anterior margin. Pronotum coarse, deeply, abundantly punctate; pubescence sparse, slightly longer between lateral tubercles of prothorax and anterior margin; with long, sparse setae. Lateral sides of prothorax with sculpture, pubescence and setae as on pronotum; lateral tubercle spiny, curved upwards. Elytra: coarse, deeply, abundantly punctate (punctures aligned in rows); pubescence sparse, forming rows placed between rows of punctures; with long, sparse setae; lateral sides slightly expanded after middle; apex together rounded. Legs: Femora and tibiae with sparse pubescence, dorsally with long, sparse setae.

Abdomen. Ventrites pubescent. Ventrite V trapezoidal, 3.3 times as long as IV, depressed at center of distal third; apex concave.

###### Type material.

Holotype female: PARAGUAY, Canindeyú: Carapa, (54°23'W, 24°22'S), 22.XI.2003, U. Drechsel col. (MZSP).

###### Dimensions in mm (female).

Total length, 4.20; length of prothorax at center, 0.85; anterior width of prothorax, 0.85; posterior width of prothorax, 0.80; largest width of prothorax, 1.05; humeral width, 1.20; elytral length, 2.95.

###### Etymology.

Latin, *concolor* = of the same color. Relating to the uniform color of the body.

###### Remarks.

*Mimasyngenes concolor* sp. n. is similar to *Mimasyngenes icuapara* Galileo & Martins, 1996, but differs mainly by the spiny lateral tubercle of prothorax being notably smaller, and by antennomere III shorter than IV. In *Mimasyngenes icuapara* the lateral tubercle of prothorax is distinctly longer and antennomere III is about as long as IV.

*Mimasyngenes concolor* can be included in the alternative of couplet “5”, from Clarke (2007) (modified):

**Table:** 

5	Lateral tubercle simple, with blunt tooth. Bolivia, Brazil (Maranhão, Pernambuco, Goiás), Argentina	***Mimasyngenes lineatipennis* Breuning, 1950**
–	Lateral tubercle with curved spine	**5**’
5’(5)	Pronotum and elytra finely punctate. Brazil (São Paulo), Bolivia	***Mimasyngenes quiuira* Galileo & Martins, 1996**
–	Pronotum and elytra coarsely punctate. Paraguay	***Mimasyngenes concolor* sp. n.**

#### Aerenicini Lacordaire, 1872

##### Recchia drechseli sp. n.

Taxon classificationAnimaliaColeopteraCerambycidae

http://zoobank.org/F3B626CF-F8B2-43F9-9D56-65D072A9ABCC

Figs 7 , 8 , 9 , 10

###### Description.

Integument dark-brown; basal two-thirds of antennomeres III–XI, mostly metatarsomere I, and basal two-thirds of tarsomeres V reddish-brown; meso- and metatibiae mostly brown.

Head. Frons trapezoidal, microsculptured, moderately fine, abundantly punctate (mainly towards clypeus); pubescence abundant, yellowish-brown, not obliterating integument (slightly whitish on some areas); with long, abundant setae. Antennal tubercles microsculptured, sparse, finely punctate (punctures sparser towards apex); pubescence as on frons. Coronal suture distinct from clypeus to anterior edge of prothorax. Vertex moderately fine, abundantly punctate; pubescence somewhat denser than on frons. Area behind eyes densely pubescent from upper eye lobes to about middle of lower eye lobes; from middle of lower eye lobes to its apex with narrow band of pubescence close to eyes, and glabrous towards anterior edge of prothorax. Genae sparsely pubescent towards eyes, glabrous towards apex. Distance between upper eye lobes equal to 0.3 times the length of scape; distance between lower eye lobes, in frontal view, equal to 0.5 times the length of scape. Antennae as long as 1.9 times the elytral length; reaching elytral apex at base of antennomere VIII; scape and pedicel with long setae throughout; antennomere III with long, moderately abundant setae on ventral side, with moderately short, abundant setae on basal half of dorsal side; antennomeres IV–XI ventrally with long, sparse setae (sparser towards distal antennomeres); antennal formula based on antennomere III: scape = 1.40; pedicel = 0.21; IV = 0.88; V = 0.88; VI = 0.83; VII = 0.81; VIII = 0.74; IX = 0.71; X = 0.62; XI = 0.64.

Thorax. Prothorax cylindrical, slightly longer than wide; lateral sides distinctly narrower at basal third. Pronotum moderately coarse, abundantly punctate (most punctures obliterated by pubescence); pubescence yellowish-white, except for a large “V-like” central area and lateral sides with yellowish-brown pubescence; with long, sparse setae. Pubescence of lateral sides of prothorax yellowish-brown; pubescence close to pronotum, gradually more yellowish-white towards prosternum. Pro- and mesosternum mostly with dark-brown pubescence. Mesepisternum with dark-brown pubescence on half close to mesosternum, yellowish-white on half close to humerus. Mesepimeron with yellowish-white pubescence. Metepisternum and lateral sides of metasternum with dense, yellowish-white pubescence; remaining surface of metasternum with pubescence less dense. Elytra: coarse, abundantly punctate on basal third, gradually finer, sparser towards apex; pubescence yellowish-white (more yellowish on some areas), not forming distinct drawing; with long, sparse setae; apex individually rounded. Legs: pubescence on femora distinctly not obliterating integument.

Abdomen. Ventrites with yellowish-brown pubescence, interspersed with long, sparse setae. Ventrite V trapezoidal, 1.4 times as long as IV; apex rounded.

###### Type material.

Holotype male: PARAGUAY, Canindeyú: Armisticio (54°32'W, 24°34'S), 30.XI.2009, U. Drechsel col. (MZSP).

###### Dimensions in mm (male).

Total length, 11.6; length of prothorax at center, 1.9; anterior width of prothorax, 1.9; posterior width of prothorax, 1.9; humeral width, 2.7; elytral length, 8.3.

###### Etymology.

The species is named for Ulf Drechsel, collector of the holotype.

###### Remarks.

*Recchia drechseli* sp. n. differs from *Recchia goiana* Martins & Galileo, 1985 as follows: dorsal pubescence less compact; upper eye lobes wider (Fig. 10), largest width larger than basal width of scape; distance between upper eye lobes equal to about 2.3 times the largest width of one lobe. In *Recchia goiana* the dorsal pubescence is more compact, the upper eye lobes are narrower (Fig. 11), with largest width about as wide as basal width of scape, and the distance between upper eye lobes is equal to 3.0 times the largest width of one lobe. It differs from *Recchia flaveola* Martins & Galileo, 1985 mainly by the femora being darker (reddish in *Recchia flaveola*), and by the elytra without distinct contrasting areas of pubescence (present in *Recchia flaveola*).

**Figures 1–16.**
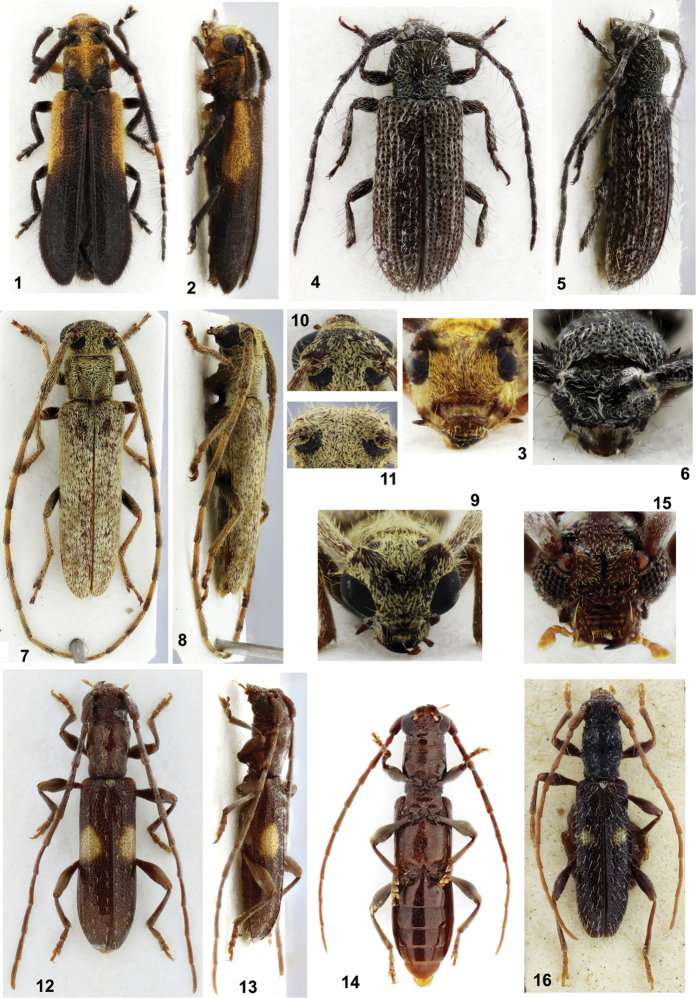
**1–3** *Eranina tomentilla*, holotype female (length, 8.0 mm): **1** Dorsal habitus **2** Lateral habitus **3** Head, frontal view **4–6** *Mimasyngenes concolor*, holotype female (length 4.2 mm): **4** Dorsal habitus **5** Lateral habitus **6** Head, frontal view **7–10** *Recchia drechseli*, holotype male (11.6 mm): **7** Dorsal habitus **8** Lateral habitus **9** Head, frontal view **10** Upper eye lobes **11** *Recchia goiana*, male, upper eye lobes **12–15** *Microibidion bimaculatum*: **12** Holotype male (5.7 mm), dorsal view **13** Holotype male, lateral view **14** Paratype female (6.6 mm), ventral view **15** Holotype male, head, frontal view **16** *Microibidion exculptum*, paratype male, dorsal view.

*Recchia drechseli* can be included in the alternative of couplet “19”, from Martins and Galileo (1998) (translated; modified):

**Table:** 

19(18)	Integument dark-brown on femora and pro- and mesosternum	**19**’
–	Integument reddish on femora and pro- and mesosternum. Brazil (Goiás, Federal District, Mato Grosso do Sul, Minas Gerais, São Paulo), Paraguay	***Recchia flaveola* Martins & Galileo, 1985**
19’(19)	Largest width of upper eye lobes about as wide as basal width of scape; distance between upper eye lobes is equal to 3.0 times the largest width of one lobe. Bolivia, Brazil (Goiás, Mato Grosso, Mato Grosso do Sul, São Paulo), Paraguay	***Recchia goiana* Martins & Galileo, 1985**
–	Largest width of upper eye lobes larger than basal width of scape; distance between upper eye lobes equal to about 2.3 times the largest width of one lobe. Paraguay	***Recchia drechseli* sp. n.**

#### Neoibidionini Monné, 2012

##### Microibidion bimaculatum sp. n.

Taxon classificationAnimaliaColeopteraCerambycidae

http://zoobank.org/F92DA9A9-C03F-4EEB-8238-16EDC9E7C10B

Figs 12 , 13 , 14 , 15

###### Description.

Male. Integument dark-brown, except for: palpi reddish; large, yellowish, sub-rounded spot on each elytron near middle, not reaching lateral side and suture.

Head. Frons transverse, coarse, abundantly, confluently punctate; pubescence yellowish, sparse, absent on narrow band around coronal suture. Antennal tubercles elevated, with sculpture and pubescence as on frons. Coronal suture distinct from clypeus to about anterior edge of eyes. Vertex moderately fine, densely, confluently punctate; pubescence sparser than on frons. Area behind eyes coarse, sparsely punctate; pubescence very sparse. Genae fine, abundantly punctate, with sparse short setae. Distance between upper eye lobes equal to 0.60 times the length of scape; distance between lower eye lobes, in frontal view, equal to 0.85 times the length of scape. Antennae as long as 1.8 times the elytral length; reaching elytral apex about apex antennomere IX; scape, pedicel and antennomeres with withish-yellow pubescence; antennomeres VII–XI somewhat curved (mainly VII–VIII); antennal formula based on antennomere III: scape = 0.76; pedicel = 0.28; IV = 0.67; V = 0.88; VI = 0.88; VII = 0.88; VIII = 0.85; IX = 0.82; X = 0.73; XI = 0.79.

Thorax. Prothorax narrower at base than anteriorly; with constriction at middle of basal half. Pronotum moderately coarse, sparsely punctate; disc with three small tubercles about middle (central more conspicuous); pubescence moderately yellowish, sparse, except for three large longitudinal glabrous areas (central longest). Lateral side of prothorax sparsely punctate; with short, very sparse setae. Pubescence on metepisterna and metasternum abundant, but not dense. Scutellum with dense, yellowish pubescence. Elytra: moderately coarse, abundantly punctate; nearly all punctures with small, fine setae; with sparse, thick, yellow, moderately long setae, somewhat aligned in three rows on basal two-thirds; apex individually rounded. Legs: pubescence on femora yellowish-brown, distinctly not obliterating integument.

Abdomen. Ventrites with pubescence as on lateral side of metasternum. Ventrite V about as long as IV; apex truncate.

Paratype female. Antennae as long as 1.5 times elytral length; slightly surpassing elytral apex. Ventrite V trapezoidal; about as long as IV; apex rounded.

###### Type material.

Holotype male: PARAGUAY, Presidente Hayes: Lolita (Laguna Yaragui, 59°37'W, 23°05'S), I.2005, U. Drechsel col. (MZSP). Paratype female: PARAGUAY, Alto Parana: Estancia Dimas (55°13'W, 25 33'S), II.2005, U. Drechsel col. (MZSP).

###### Dimensions in mm (male/female).

Total length, 5.70/6.60; length of prothorax at center, 1.20/1.30; anterior width of prothorax, 0.85/0.95; posterior width of prothorax, 0.75/0.85; humeral width, 1.15/1.30; elytral length, 3.70/4.10.

###### Etymology.

Latin, *bi* = two; *maculatus* = spotted. Relating to the two spots on elytra.

###### Remarks.

*Microibidion bimaculatum* sp. n. is similar to *Microibidion exculptum* Martins, 1962, but differs as follows: antennae dark-brown; antennomeres somewhat thicker in both sexes; antennomeres X and XI about as long as IV; basal antennomeres without long, sparse setae on ventral side; thick setae on basal two-thirds of elytra aligned in three rows. In *Microibidion exculptum* (Fig. 16) the antennae are reddish, the antennomeres are slender, antennomeres X and XI are shorter than IV, the basal antennomeres have long, sparse setae on ventral side, and the thick setae on basal two-thirds of elytra are aligned in 4/5 rows.

*Microibidion bimaculatum* can be included in the alternative of couplet “3”, from Martins (2009) (translated):

**Table:** 

3(2)	Head, prothorax and elytra reddish; upper eye lobes with two rows of ommatidia. Brazil (Espírito Santo to Santa Catarina)	***Microibidion muticum* (Martins, 1962)**
–	Head, prothorax and elytra dark-brown or black; upper eye lobes with three rows of ommatidia	**3**’
3’(2)	Antennomeres X and XI shorter than IV; basal antennomeres ventrally with long, sparse setae; thick setae on elytra aligned in 4/5 rows on basal two-thirds. Brazil (São Paulo to Rio Grande do Sul), Paraguay, Argentina (Misiones, Buenos Aires)	***Microibidion exculptum* Martins, 1962**
–	Antennomeres X and XI about as long as IV; basal antennomeres ventrally without long setae; thick setae on elytra aligned in 3 rows on basal two-thirds. Paraguay	***Microibidion bimaculatum* sp. n.**

## Supplementary Material

XML Treatment for Eranina tomentilla

XML Treatment for Mimasyngenes concolor

XML Treatment for Recchia drechseli

XML Treatment for Microibidion bimaculatum
